# Adipokines in Nonalcoholic Steatohepatitis: From Pathogenesis to Implications in Diagnosis and Therapy

**DOI:** 10.1155/2009/831670

**Published:** 2009-06-03

**Authors:** Emmanuel A. Tsochatzis, George V. Papatheodoridis, Athanasios J. Archimandritis

**Affiliations:** 2nd Department of Internal Medicine, Medical School of Athens University, Hippokration General Hospital, 115 27 Athens, Greece

## Abstract

Nonalcoholic fatty liver disease (NAFLD) is the hepatic manifestation of the metabolic syndrome and can vary from benign steatosis to end-stage liver disease. The pathogenesis of non-alcoholic steatohepatitis (NASH) is currently thought to involve a multiple-hit process with the first hit being the accumulation of liver fat which is followed by the development of necroinflammation and fibrosis. There is mounting evidence that cytokines secreted from adipose tissue, namely, adipokines, are implicated in the pathogenesis and progression of NAFLD. In the current review, we explore the role of these adipokines, particularly leptin, adiponectin, resistin, tumor necrosis factor-a, and interleukin-6 in NASH, as elucidated in experimental models and clinical practice. We also comment on their potential use as noninvasive markers for differentiating simple fatty liver from NASH as well as on their potential future therapeutic role in patients with NASH.

## 1. Introduction

Nonalcoholic fatty liver disease (NAFLD) is the hepatic manifestation of the metabolic syndrome and is closely associated with visceral obesity and insulin resistance [1–3]. The prevalence of NAFLD is 10–25% in the western world [4–6]; only in USA, an estimated number of 80 million adults are affected [7]. The term NAFLD encompasses a wide spectrum of liver histology, from simple steatosis to nonalcoholic steatohepatitis (NASH), which can progress to cirrhosis and its complications [7]. The pathogenesis of NASH is currently thought to involve a multiple step process, in which the first step is the accumulation of liver fat which is followed by the development of necroinflammation and fibrosis [8]. Distinguishing NAFLD from NASH is essential in the clinical setting, as NAFLD seems to have a benign course in the absence of co-existing liver disease, in sharp contrast to NASH, which is associated with increased morbidity and mortality and a reduced life expectancy [9, 10]. Although noninvasive clinical markers have been proposed, liver biopsy still remains the gold standard for the above distinction [11].

Cytokines play a pivotal role in pathogenesis and severity of NAFLD. In the last decade, adipose tissue has emerged as an endocrine organ with a key role in energy homeostasis, since its metabolic products, adipokines, exert local, peripheral, and central effects [12]. Obesity is currently regarded as a systemic, low-grade inflammation, in which adipose tissue and its hormones have a central role [13]. Hypertrophied adipocytes release chemokines, which recruit macrophages and a vicious cycle commences, as macrophages release inflammatory cytokines, which stimulate inflammatory and suppress anti-inflammatory adipokines [14]. In the setting of liver injury, most adipokines are also produced and secreted by hepatocytes. In the current review, we explore the role of cytokines in the clinical setting of NAFLD, in terms of pathogenesis, their use as markers of disease progression and their perspective as therapeutic targets. We particularly focus on adipokines, namely, leptin, adiponectin, and resistin, tumour necrosis factor-a (TNFa) and interleukin-6 (IL-6) and we briefly discuss the potential role of recently discovered visfatin and retinol-binding protein 4 (RBP4).

## 2. Cytokines and NAFLD—Experimental Models

### 2.1. NAFLD and NASH: Pathophysiology

The primary insult in NASH is accumulation of triglycerides in the liver, as a result of insulin resistance [7]. The main effect of insulin is to increase glucose uptake by cells through upregulation of the glucose transporters expression on the cells surface. Insulin also induces lipogenesis and inhibits lipolysis in the adipose tissue [15], while it increases the synthesis of fatty acids in the liver. Insulin resistance and the metabolic syndrome lead to defective insulin-mediated inhibition of lipolysis, mostly in visceral fat, while hyperinsulinemia results in increased hepatic synthesis of free fatty acids and decreased synthesis of apolipoprotein B-100. Thus, insulin resistance results in both increased adipose tissue lipolysis and increased hepatic lipogenesis leading to lipid accumulation in the hepatocytes, mainly in the form of triglycerides [16]. 

According to the “two-hit” hypothesis, the development of NASH requires the presence of additional pathophysiological abnormalities. The second hit is currently believed to be increased oxidative stress within the hepatocytes, which is characterized by excessive production of reactive oxygen species (ROS) [17]. Mitochondria and the cytochrome P-450 system are considered to be the major ROS generation sites in the liver [17, 18]. ROS promote progression from steatosis to steatohepatitis and fibrosis by three main mechanisms: lipid peroxidation, cytokine induction, and Fas ligand induction [19–21]. It must be noted that as the term two-hit might be too simplistic to elaborate the complex pathogenic events occurring in NASH patients, multiple-step models taking into account that lipid injury to hepatic veins have been formed [22]. In this complex setting, the role of various adipokines has been clearly elucidated.

### 2.2. Leptin

Leptin is a 167-amino acid protein discovered by positional cloning of the ob gene in 1994 [23]. Although it is considered as an anorexigenic hormone, its levels are elevated in obesity as a result of resistance to its actions [24]. In animal models, it prevents lipid accumulation in nonadipose tissues [25]. In particular in the liver, this effect is achieved by lowering the expression of sterol regulating binding protein 1 (SREBP-1) [26]. Leptin is thought to participate in both hits of NASH development. Initially, it contributes to the development of insulin resistance and subsequently steatosis. Furthermore, in the context of liver insult, leptin has a proinflammatory role and is considered to be an essential mediator of liver fibrosis [12]. In rats treated with carbon tetrachloride (CCl_4_), leptin injections have been shown to result in increased expression of procollagen-I, transforming growth factor *β*1 (TGF*β*1) and smooth muscle actin, a marker of activated hepatic stellate cells (HSCs), and eventually to increased liver fibrosis [27]. In the latter study [27], there was also a dramatic increase in serum TNFa levels after leptin injections, suggesting that leptin may amplify inflammation and independently affect the development of fibrosis [28]. Sinusoidal endothelial cells and Kupffer cells were identified as the main targets of the profibrogenic action of leptin [27]. On the other hand, leptin-resistant [29] or leptin-deficient mice [30] exhibit significantly reduced fibrogenic responses. This fibrogenic effect is accomplished through HSCs, in which leptin is a potent mitogen and apoptosis inhibitor. Activated HSCs acquire the ability to secrete leptin and further promote liver fibrosis [12].

### 2.3. Adiponectin

Adiponectin is exclusively secreted by adipocytes and is considered as an anti-inflammatory adipokine [31]. It reduces body fat, improves hepatic and peripheral insulin sensitivity, and is inversely associated with body mass index and insulin resistance [32]. In the liver, it prevents lipid accumulation by increasing *β*-oxidation of free fatty acids and/or by decreasing de novo free fatty acids within hepatocytes [33, 34]. Part of this action is accomplished through the downregulation of SREBP-1, a key regulator of fatty acid synthesis [35]. As a result of the above, adiponectin knockout mice develop more severe hepatic steatosis than wild-type mice when fed with a diet aiming to induce NASH [36]. Furthermore, adiponectin has a protective role in liver inflammation and fibrosis; in their classic experiment, Kamada et al. showed that adiponectin treatment attenuated liver fibrosis in mouse models treated with CCl_4_ and that HSCs express both adiponectin receptors (AdipoR1 and AdipoR2). Using a mouse model of endotoxin induced acute liver injury in KK-Ay obese mice, Masaki et al. found that adiponectin prevents hepatic injury by inhibiting the synthesis and/or release of TNFa [37], while Xu et al. also observed a protective effect of adiponectin in fatty liver disease in mice [38]. In another mouse model, the adiponectin resistance and sensitivity mediated by adipoR2 regulated NASH progression and this was accomplished by changing PPARa activity and ROS accumulation. The authors thus concluded that the liver adipoR2 signalling pathway could be a promising target in treating NASH [39]. Finally, adiponectin induction was associated with the protective action of saturated fat against the development of alcoholic fatty liver disease in mice [34].

### 2.4. Tumour Necrosis Factor-a

TNFa has also a central role in the development of fatty liver and subsequently NASH. Elevated circulating TNFa levels are associated with obesity and insulin resistance both in animal models and humans [40, 41]. The lipogenic action of TNFa is in part accomplished by the upregulation of SREBP-1 [34]. Furthermore, the 238 TNFa gene polymorphism was found significantly more frequently in patients with fatty liver than healthy controls [42] and the 1031C and 863A polymorphisms in NASH than fatty liver [43]. Moreover, elevated TNFa production has been observed in cultures of peripheral blood cells collected from obese patients with NASH [44]. Mice genetically deficient in TNF receptor I have proved resistant to NASH induced by two different diets [45, 46], while treatment of leptin-deficient mice with TNFa antibodies improved hepatic insulin resistance and fatty liver [47]. It was further suggested that TNFa profibrotic action is mediated through Kupffer cells activation [46].

### 2.5. Resistin

Resistin is a recently discovered adipokine, secreted by adipose tissue and macrophages [48]. A number of rodent studies suggest that the major target of resistin action is the liver, causing hepatic insulin resistance, with additional effects on skeletal muscles and adipose tissue [49–52]. In skeletal muscle, it seems to reduce the uptake and metabolism of free fatty acids, thus contributing to insulin resistance [53]. Moreover, there is evidence that resistin treatment markedly induces the gene expression of suppressor of cytokine signalling 3 (SOCS3), a known inhibitor of insulin signalling [54]. Most importantly, there is evidence of a proinflammatory resistin action, as it stimulates TNFa and IL-12 in macrophages by a nuclear factor (NF)-kappaB-dependent pathway [55] and regulates the secretion of IL-6 and IL-1*β* [56]. Finally, evidence from one study suggests that resistin has a proinflammatory action in HSCs, thus participating in liver fibrogenesis [57].

### 2.6. Interleukin-6

The issue seems to be more complex with IL-6, a cytokine secreted by adipocytes, immune, and endothelial cells. Initial reports supported a hepatoprotective action of IL-6 in steatotic livers, by suppressing oxidative stress and preventing mitochondrial dysfunction [58, 59]. Moreover, IL-6 has a hepatoprotective effect in ischaemic preconditioning models [60] and is important for survival after partial hepatectomy in mice [61]. However, this seems to be a paradoxical effect of short- and long-term IL-6 exposure, as the latter may sensitize the liver to injury and apoptotic cell death [62]. It remains to be elucidated whether elevated IL-6 levels in chronic liver injury contribute to inflammation or represent an anti-inflammatory response.

### 2.7. Other Cytokines

RBP4 is predominantly secreted by visceral adipose tissue and is elevated in the insulin-resistant state [63]. A pathogenetic link of elevated adipose and serum levels of RBP4 with insulin resistance and diabetes has been found in both animal and human studies [63–65]. Moreover, RBP4 levels were associated with the inflammatory response in obese individuals [66, 67]. 

Visfatin is a recently discovered adipokine that exerts insulin-mimicking effects, by activating the insulin receptor in a manner distinct from that of insulin [68]. There is evidence that supports a possible coregulation with IL-6, as visfatin induces the production of IL-6 in human CD4 [59], whereas IL-6 negatively regulates visfatin gene expression in 3T3-LI adipocytes [60]. There are currently no studies to support a pro- or anti-inflammatory visfatin action.

## 3. Cytokines and NASH—Human Studies

The above observations in experimental models of steatosis and liver injury, elucidating a potential association of various cytokines in the development of NASH, have resulted in their evaluation in patients with NASH. 

The first adipokine to be addressed was leptin, with contradictory results. Leptin levels were initially found to be significantly higher in 47 NASH patients than 47 controls and correlated with the severity of hepatic steatosis but not of necroinflammation or fibrosis [69]. Three more studies, comprising of 88, 26, and 25 patients, respectively, [70–72], failed to show any significant difference in leptin levels between NASH and controls or any independent association with liver fibrosis. In a recent study, we showed that leptin levels are significantly higher in NASH patients than patients with chronic viral hepatitis and correlate with more severe fibrosis in univariate analysis [73]. Other studies have also reported conflicting results [74]. Thus, it is doubtful whether leptin is upregulated in patients with NASH and larger studies with homogenous population and carefully matched healthy controls are needed. For the time being, leptin cannot be used as a noninvasive marker for the diagnosis of NASH.

Results have been more promising with adiponectin, as its levels are lower in patients with NAFLD/NASH than controls [75]. Furthermore, important steps have been taken towards its use as a noninvasive marker for the differentiation between NAFLD and NASH. Hui et al. showed that adiponectin was significantly reduced in subjects with NASH compared to age, BMI, and sex matched healthy controls or compared to patients with simple steatosis [76]. The authors found that the combined use of adiponectin and homeostasis model assessment for insulin resistance (HOMA-IR) for differentiating NASH from fatty liver had an area under the curve value of 0.79. Attempting to set a discriminating adiponectin level, they found that 77% of patients with NASH had adiponectin <10 *μ*g/mL and HOMA-IR >3, compared to 33% of patients with simple steatosis. In another study comprising of 66 patients with early NASH stages (Brunt 1-2) and 19 with simple steatosis, the combined evaluation of serum adiponectin, HOMA-IR, and serum collagen type IV had 94% sensitivity and 74% specificity for the prediction of early NASH [77]. When adiponectin was used alone, the area under the receiver operating characteristic (ROC) curve was 0.765 for the above prediction. Finally, in 101 obese patients with NASH, a biomarker panel comprised of cleaved cytokeratin 18 (CK-18), adiponectin, and resistin had 95% sensitivity and 70% specificity for diagnosis [78]. All three mentioned studies have yielded promising results towards a noninvasive method of diagnosing NASH. The measurement of high molecular weight adiponectin levels, which are thought to better correlate with steatosis than the total adiponectin levels [79] and the validation of these markers in larger cohorts would be crucial steps towards the widespread use of these biomarker panels.

Apart from adiponectin serum levels, the hepatic and adipose expression of its receptors have been measured with conflicting results. In the largest study, adiponectin receptors were underexpressed in visceral fat but overexpressed in liver and positively associated with liver damage [80]. The authors concluded that the liver overexpression possibly constituted a compensatory response to hypoadiponectinemia. Two smaller studies, however, yielded contradictory results, as they found that hepatic expressions of adiponectin mRNA and adipoR2 receptor were significantly lower in NASH patients compared with patients with fatty liver [81, 82]. In a recent study, single nucleotide adiponectin polymorphisms (SNPs) were studied in 70 nonobese, nondiabetic, normolipidemic NAFLD patients, and 70 healthy controls. Interestingly, it was shown that two at-risk adiponectin SNPs (45TT and 276GT) were significantly more prevalent in NAFLD than in the general population, and that they were associated with severity of liver disease independently of adiponectin serum levels [83]. As genetic testing is due to become readily available, future use of SNPs as noninvasive disease markers may be feasible.

Results are contradictory in the case of TNFa and might reflect different study populations or the lack of adjustment for several factors that may influence its serum levels. Hui et al. showed that although TNFa levels significantly differed between NAFLD and controls, no such difference existed between NASH and NAFLD [76]. In a small study of 23 patients with NASH, 21 with NAFLD and 18 controls, serum TNFa and soluble TNF receptor 1 were significantly increased in NASH compared to both NAFLD and controls [84]. Finally, no significant difference of serum TNFa levels was found between a population of nonobese nondiabetic NASH patients and matched controls [72].

Resistin is inadequately studied in NASH, and clearly more data is needed for solid conclusions. In a study of 80 ethnic Chinese patients with NAFLD, serum resistin levels were associated neither with presence of NASH nor with its severity [85]. In a smaller study of 45 NAFLD patients and 50 controls, serum TNFa but not resistin was independently associated with fibrosis in NASH [86]. On the other hand, resistin levels were associated with fibrosis severity in a recent study from our group [73], while serum resistin levels were higher in NAFLD than controls and positively correlated with liver inflammation in an Italian study [87]. In the largest study so far, resistin was part of the biomarker panel for the noninvasive diagnosis of NASH [78].

Regarding the remaining cytokines, data is yet scarce and confined to small patient numbers. IL-6 has gained attention lately, after a small study of 35 patients (NASH: 18, NAFLD: 10, controls: 7), in which IL-6 liver expression was markedly increased in NASH patients and positively correlated with inflammation and fibrosis [88]. In a study of 36 morbidly obese patients and 12 healthy controls, IL-6 was an independent predictor of steatosis and NASH [89]. Finally, serum IL-6 was found significantly elevated in NAFLD than controls, even after correction for age, sex, and BMI [90]. RBP4 serum levels were higher both in diabetic and nondiabetic NAFLD patients compared to controls, but no liver biopsy was performed and, therefore, histological correlations were not available [91]. Finally, although serum visfatin was lower in NASH than NAFLD patients and healthy controls, it was not independently associated with presence of NASH [86], while its levels positively correlated with portal inflammation [92]. The potential role of different adipokines in NAFLD is summarized in Table 1.

## 4. Therapeutic Implications

As it has been clearly elucidated, adipokines are key players in the pathogenesis and progression of NAFLD. As there is no effective specific therapy for NASH, the use of drugs directly targeting adipokines seems a reasonable option. To date, few drugs have been tested towards that direction. 

Pentoxifylline, a nonspecific TNFa inhibitor, was used in a pilot study including 20 patients with NASH at a daily dose of 1200 mg [93]. A significant decrease of serum transaminases was noted after 12 months, but no follow-up histology was available. A significant proportion of patients could not tolerate the drug due to nausea and gastrointestinal symptoms. Histological improvement was documented in 9 patients treated with a lower dose of pentoxifylline in another recent study, in which adverse effects were not reported [94]. Infliximab is a chimeric monoclonal antibody that selectively and potently blocks TNFa. In experimental models, it improves NASH lesions, and, therefore, its trial in humans could be of interest [95]. The detrimental effect of infliximab in patients of alcoholic hepatitis is a potential concern [96].

Adiponectin exerts hepatoprotective actions in animal models, and its administration could prove beneficial in subjects with NASH. Pioglitazone, which significantly improves NASH lesions, also increases adiponectin levels providing indirect evidence of a potential adiponectin effect [97].

Finally, a humanized IL-6 receptor antibody, tocilizumab, has been developed and successfully used in patients with rheumatoid arthritis [98]. Its future trial in patients with NASH would be helpful and welcome.

## 5. Conclusions

NAFLD represents a spectrum of disease from benign fatty liver to NASH and even cirrhosis. The disease seems to progress through a two- or even multiple-hit process, with successive liver insults leading from fatty infiltration to inflammation and fibrosis. The interplay of various adipokines, the most important of which are leptin, adiponectin, TNFa, resistin, and IL-6, has a key role in this process. Clinical studies suggest that serum levels of the above cytokines differ among patients with NAFLD/NASH and healthy controls. As liver biopsy remains the gold standard for diagnosing NASH, the use of adipokines as noninvasive diagnostic test is of interest. To date, adiponectin has yielded the most promising results, but its use should be validated in larger cohorts of patients. Finally, the use of drugs that specifically target adipokines remains an attractive possibility and a future challenge.

## Figures and Tables

**Table 1:** The role of different adipokines in NAFLD.

Cytokine	Experimental models	Clinical studies
Leptin	Proinflammatory	Not elevated in NAFLD/NASH
	HSCs activation	No association with histology
Adiponectin	Anti-inflammatory	Lower in NAFLD than controls
	Hepatoprotective	Inversely correlates with fibrosis
Resistin	Proinflammatory	Elevated in NASH
		Possible association with fibrosis
TNF-a	Proinflammatory	Elevated in NAFLD/NASH
	Lipogenic	Correlates with fibrosis
IL-6	Uncertain	Insufficient evidence
Visfatin	Uncertain	Insufficient evidence
RBP-4	Proinflammatory	Insufficient evidence

TNF-a: tumour necrosis factor-a; IL-6: interleukin-6; RBP-4: retinol-binding protein-4; HSCs: hepatic stellate cells; NAFLD: nonalcoholic fatty liver disease; NASH: nonalcoholic steatohepatitis.
